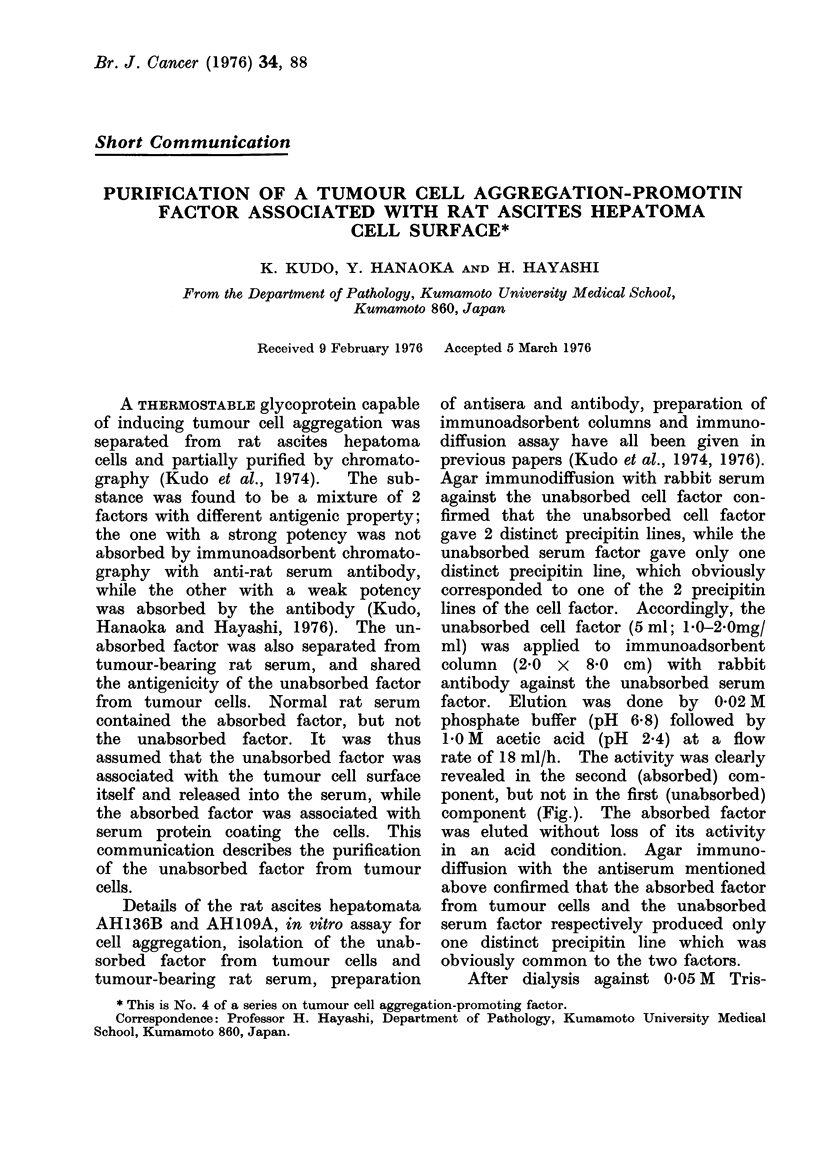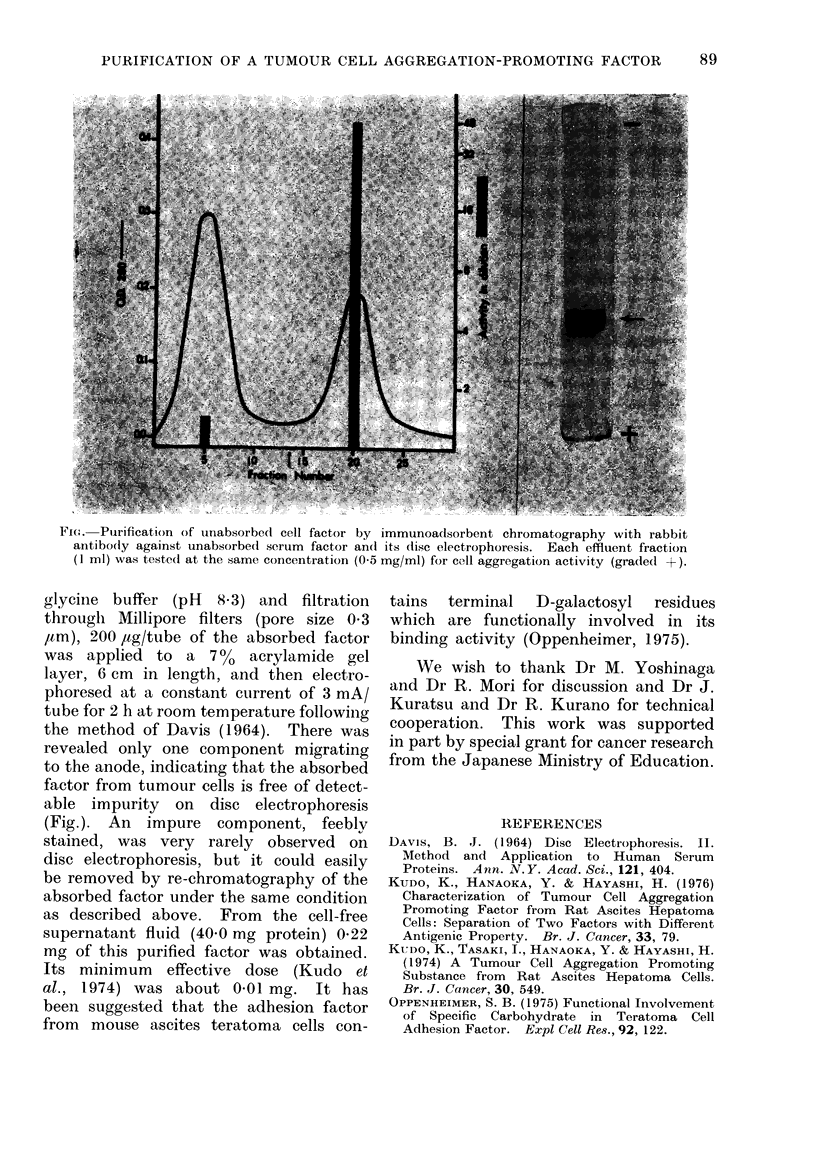# Purification of a tumour cell aggregation-promotin factor associated with rat ascites hepatoma cell surface.

**DOI:** 10.1038/bjc.1976.126

**Published:** 1976-07

**Authors:** K. Kudo, Y. Hanaoka, H. Hayashi

## Abstract

**Images:**


*Br. J. Cancer* (1976) **34**, 88

### *Short Communication*

### PURIFICATION OF A TUMOUR CELL AGGREGATION-PROMOTIN FACTOR ASSOCIATED WITH RAT ASCITES HEPATOMA CELL SURFACE*

K. KUDO, Y. HANAOKA AND H. HAYASHI

*From the Department of Pathology, Kumamoto University Medical School, Kumamoto* 860, *Japan*

Received 9 February 1976 Accepted 5 March 1976

A THERMOSTABLE glycoprotein capable of inducing tumour cell aggregation was separated from rat ascites hepatoma cells and partially purified by chromatography (Kudo *et al.*, 1974). The substance was found to be a mixture of 2 factors with different antigenic property; the one with a strong potency was not absorbed by immunoadsorbent chromatography with anti-rat serum antibody, while the other with a weak potency was absorbed by the antibody (Kudo, Hanaoka and Hayashi, 1976). The unabsorbed factor was also separated from tumour-bearing rat serum, and shared the antigenicity of the unabsorbed factor from tumour cells. Normal rat serum contained the absorbed factor, but not the unabsorbed factor. It was thus assumed that the unabsorbed factor was associated with the tumour cell surface itself and released into the serum, while the absorbed factor was associated with serum protein coating the cells. This communication describes the purification of the unabsorbed factor from tumour cells.

Details of the rat ascites hepatomata AH136B and AH109A, *in vitro* assay for cell aggregation, isolation of the unabsorbed factor from tumour cells and tumour-bearing rat serum, preparation of antisera and antibody, preparation of immunoadsorbent columns and immunodiffusion assay have all been given in previous papers (Kudo *et al.*, 1974, 1976). Agar immunodiffusion with rabbit serum against the unabsorbed cell factor confirmed that the unabsorbed cell factor gave 2 distinct precipitin lines, while the unabsorbed serum factor gave only one distinct precipitin line, which obviously corresponded to one of the 2 precipitin lines of the cell factor. Accordingly, the unabsorbed cell factor (5 ml; 1·0–2·0mg/ml) was applied to immunoadsorbent column (2·0 × 8·0 cm) with rabbit antibody against the unabsorbed serum factor. Elution was done by 0·02 M phosphate buffer (pH 6·8) followed by 1·0 M acetic acid (pH 2·4) at a flow rate of 18 ml/h. The activity was clearly revealed in the second (absorbed) component, but not in the first (unabsorbed) component (Fig.). The absorbed factor was eluted without loss of its activity in an acid condition. Agar immunodiffusion with the antiserum mentioned above confirmed that the absorbed factor from tumour cells and the unabsorbed serum factor respectively produced only one distinct precipitin line which was obviously common to the two factors.

After dialysis against 0·05 M Tris-

* This is No. 4 of a series on tumour cell aggregation-promoting factor.

Correspondence: Professor H. Hayashi, Department of Pathology, Kumamoto University Medical School, Kumamoto 860, Japan.

FIG.—Purification of unabsorbed cell factor by immunoadsorbent chromatography with rabbit antibody against unabsorbed serum factor and its disc electrophoresis. Each effluent fraction (1 ml) was tested at the same concentration (0·5 mg/ml) for cell aggregation activity (graded +).

glycine buffer (pH 8·3) and filtration through Millipore filters (pore size 0·3 $\mu$m), 200 $\mu$g/tube of the absorbed factor was applied to a 7% acrylamide gel layer, 6 cm in length, and then electrophoresed at a constant current of 3 mA/tube for 2 h at room temperature following the method of Davis (1964). There was revealed only one component migrating to the anode, indicating that the absorbed factor from tumour cells is free of detectable impurity on disc electrophoresis (Fig.). An impure component, feebly stained, was very rarely observed on disc electrophoresis, but it could easily be removed by re-chromatography of the absorbed factor under the same condition as described above. From the cell-free supernatant fluid (40·0 mg protein) 0·22 mg of this purified factor was obtained. Its minimum effective dose (Kudo *et al.*, 1974) was about 0·01 mg. It has been suggested that the adhesion factor from mouse ascites teratoma cells contains terminal D-galactosyl residues which are functionally involved in its binding activity (Oppenheimer, 1975).

We wish to thank Dr M. Yoshinaga and Dr R. Mori for discussion and Dr J. Kuratsu and Dr R. Kurano for technical cooperation. This work was supported in part by special grant for cancer research from the Japanese Ministry of Education.

### REFERENCES

DAVIS, B. J. (1964) Disc Electrophoresis. II. Method and Application to Human Serum Proteins. *Ann. N.Y. Acad. Sci.*, **121**, 404.

KUDO, K., HANAOKA, Y. & HAYASHI, H. (1976) Characterization of Tumour Cell Aggregation Promoting Factor from Rat Ascites Hepatoma Cells: Separation of Two Factors with Different Antigenic Property. *Br. J. Cancer*, **33**, 79.

KUDO, K., TASAKI, I., HANAOKA, Y. & HAYASHI, H. (1974) A Tumour Cell Aggregation Promoting Substance from Rat Ascites Hepatoma Cells. *Br. J. Cancer*, **30**, 549.

OPPENHEIMER, S. B. (1975) Functional Involvement of Specific Carbohydrate in Teratoma Cell Adhesion Factor. *Expl Cell Res.*, **92**, 122.